# A Cadaveric Case Report of a Double Right Coronary Artery and Its Clinical Implications

**DOI:** 10.7759/cureus.48578

**Published:** 2023-11-09

**Authors:** Axel B Lichtenberg, Kamal A Abouzaid, Ahmad Y Karim, Vanessa Cornelio, Mohammad Algoul, Ahmad Imam

**Affiliations:** 1 Department of Anatomical Sciences, William Carey University College of Osteopathic Medicine, Hattiesburg, USA

**Keywords:** cadaveric case report, carina-like septum, anatomy of anomalous coronary artery, double right coronary artery, right coronary artery (rca)

## Abstract

A double right coronary artery is one of the rarest variations of the coronary arterial system. This case report presents a unique variation: a right coronary and an accessory coronary artery arise from a single ostium within the right aortic sinus. The two vessels appeared externally to have a common trunk but were partitioned internally by a carina-like septum. This report underscores the importance of understanding the embryological development of coronary arteries and recognizing potential variations. It discusses the specific variation observed in this case and its clinical implications. The aim is to contribute to the limited literature on this condition and highlight the importance of recognizing and managing such anomalies in clinical procedures.

## Introduction

The human heart undergoes a complex and precise process of development. According to Willhelm His, the embryological development of the heart begins around the third week of gestation with the formation of two endocardial tubes, which soon fuse to form a primitive heart tube. This heart tube then undergoes a process of looping, septation, and further differentiation to form the four chambers of the heart and associated structures [1]. There is some controversy regarding the development of normal coronary arteries, but the literature indicates that coronary arteries are derived from multiple different cell types and embryological sources [2,3].

Building on this understanding, it is essential to recognize the coronary arteries' specific origin and formation process. The coronary arteries originate from the sinus venosus, initially forming as endothelial plexuses around the aortic root. Through a complex remodeling process, they eventually form the mature coronary system [4]. According to a recent review, anomalous coronary arteries result from deviations occurring in the embryological progenitor cells, the interaction between coronary vessels and myocardium, and the process connecting the coronary arteries to the aorta [2]. Anomalies of coronary arteries are not uncommon. The prevalence of these anomalies is reported to be around 1-5.6% in the general population [5,6]. 

In this case report, we identified a rare variation where the right coronary artery (RCA) was associated with an accessory RCA (aRCA), also known as a double RCA anomaly. Our findings contribute to the current literature on this condition as one of the few cadaveric case reports that fully display images of both the inner carina-like septum and the external course of the double RCA anomaly. This report emphasizes the importance of expanding our knowledge of coronary artery variations to avoid potential complications during clinical procedures. 

## Case presentation

**Figure 1:**
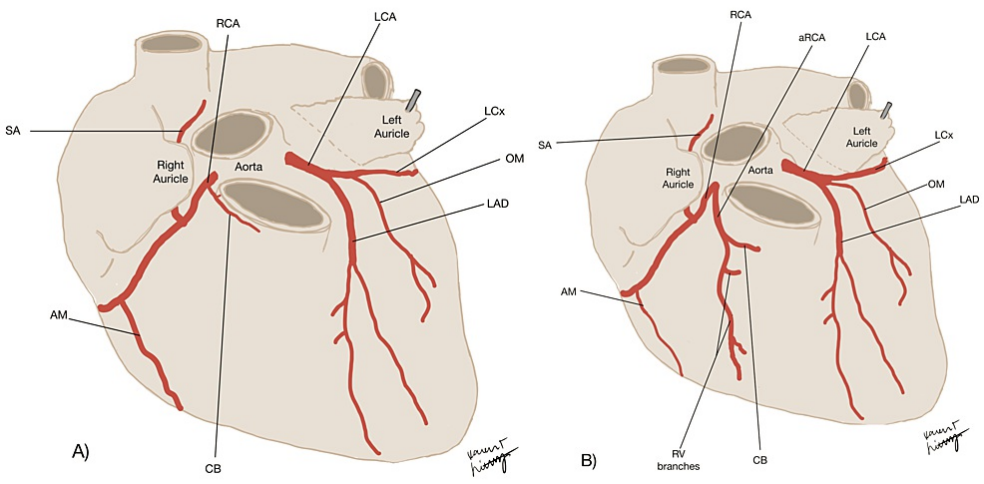
A schematic representing and comparing normal coronary arteries (A) and the variation seen in this case (B). AM: acute marginal branch of RCA; aRCA: accessory right coronary artery; CB: conus branch of RCA; LAD: left anterior descending artery, LCA: left coronary artery; LCx: left circumflex branch of LCA; OM: obtuse marginal branch of LCA; RCA: right coronary artery; RV branches: right ventricular branches of aRCA; SA: sinoatrial nodal branch of RCA. Image credit: Karen Lichtenberg

During the summer prosection course at William Carey University College of Osteopathic Medicine, students dissected a 51-year-old male cadaver obtained from the University of Southern Alabama Anatomical Gift Program. The cause of death, as stated on the death certificate, was liver disease. An intriguing finding emerged as the coronary vessels were dissected; two vessels seemed to arise from the right coronary sinus as a main RCA and an additional aRCA. The branches were meticulously dissected, studied, and documented for further analysis. Schematics were produced to illustrate the variations found in this case (Figure 1). 

**Figure 2:**
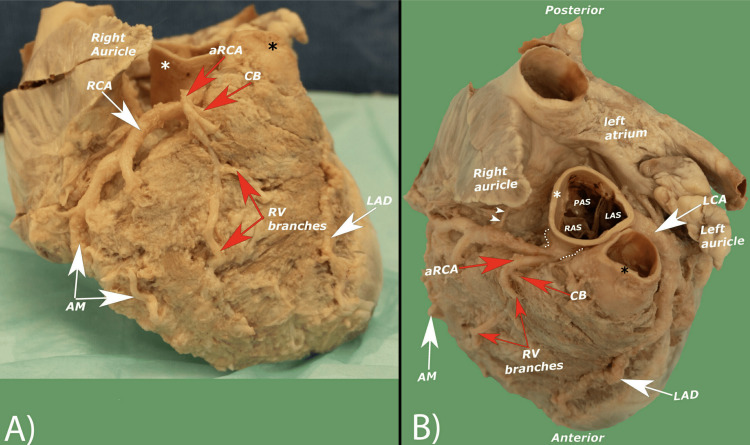
The heart in anatomical position (A) and the heart from a superior view (B) displaying the variation seen in this case. AM: acute marginal branch of RCA; aRCA: accessory right coronary artery; CB: conus branch of RCA; LAD: left anterior descending artery; LCA: left coronary artery; RCA: right coronary artery; RV branches: right ventricular branches of aRCA; white asterisk: aorta; black asterisk: pulmonary trunk; white arrow heads: sinoatrial nodal branch; dotted line: common trunk.

Originating from what externally appeared as a very short common trunk, the main RCA has the normal expected course within the coronary sulcus. Along its path, it provided branches such as the sinoatrial artery, right atrial artery, and acute marginal artery and continued as the posterior descending artery, which aligned with the expected distribution pattern of the RCA. From the common trunk, a second artery was identified as an accessory RCA that descended on the infundibulum and the anterior wall of the right ventricle, gave rise to a conus branch, and terminated as right ventricular branches (Figure 2). 

**Figure 3:**
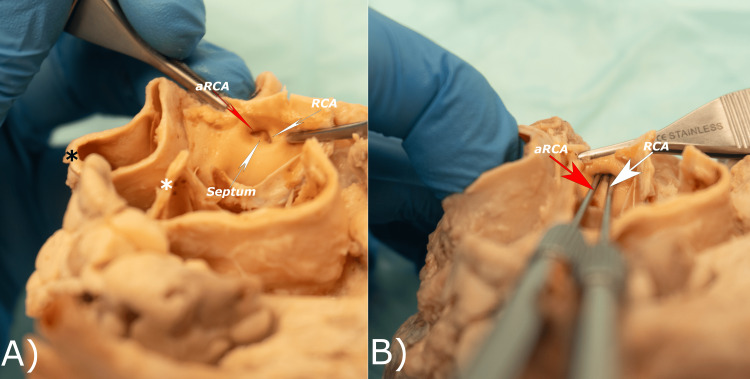
The heart with the ascending aorta cut to show the right aortic sinus with the main ostium and openings of the RCA and aRCA, and the inner septum separating them (left). Probes have been placed into the openings (right) to show the patency of the arteries. aRCA: accessory right coronary artery; RCA: right coronary artery; black asterisk: pulmonary trunk; white asterisk: aorta.

An incision was made to remove a part of the ascending aorta to the level of the aortic sinuses, which allowed us to better visualize the ostia of the coronary vessels. The ostium for the left coronary artery (LCA) was found to be at a level slightly superior to the level of the ostium of the RCA. A second incision was then made in the ascending aorta between the valve leaflets (Figure 3). Consequently, the ostia of the coronary vessels were better exposed. A carina-like septum within the right coronary ostium divided this main ostium into two smaller ostia that continued as the RCA and the aRCA. 

**Figure 4:**
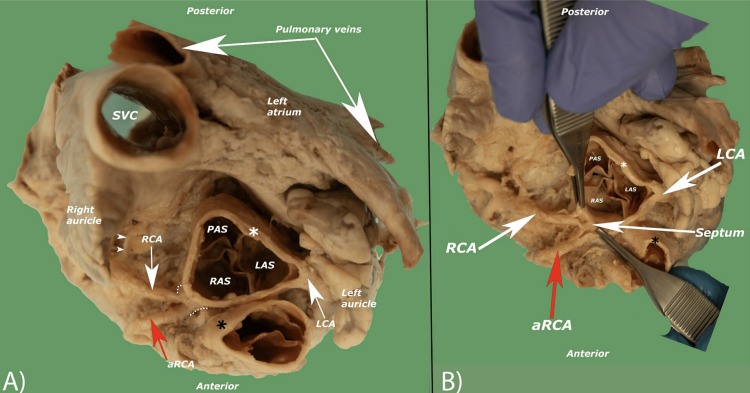
A superior view of the heart showing the common origin of the two vessels (A). The common origin was deroofed to show the inner septum (B). AM: acute marginal branch of RCA; aRCA: accessory right coronary artery; CB: conus branch of RCA; LAD: left anterior descending artery; LCA: left coronary artery; RCA: right coronary artery; RV branches: right ventricular branches of aRCA; white asterisk: aorta; black asterisk: pulmonary trunk; dotted line: common origin.

Although the beginning of the two vessels appeared as a short common trunk (0.49 mm) externally, further dissection of the common trunk's lumen revealed that the carina-like septum divided the trunk internally into the main RCA and the aRCA (Figure 4). The septum is 1.2 mm from the endothelial surface of the aorta. The diameters of the RCA and the aRCA, measured at their proximal origins, were 1.8 mm and 1.6 mm, respectively.

## Discussion

A double RCA is one of the rarest coronary artery anomalies, with a prevalence of 0.01-0.07% [7]. The first report of its existence was in 1956 [8]. There is still some debate about the naming convention for double RCAs [9]. However, it is known that a double RCA can arise from a single ostium and split into two branches after a variable short distance from a proximal common trunk, or it can originate from two separate ostia [7]. In this report, we named the RCA that follows the classical course in the coronary sulcus as the “main coronary artery” and referred to the additional RCA as the “accessory coronary artery.”

Almost all reports of a double RCA are found incidentally on coronary angiography [7,10]. While most angiographic cases of double RCA describe an aRCA with a course similar to what we have observed in the present case, this report may be the only cadaveric report that provides additional anatomical insights, including photographic depiction of the course of the arteries and the shape of the internal septum. Interestingly, unlike the three previously reported cadaveric cases, which did not align with the typical angiographic descriptions, our case more closely mirrors the anatomical details frequently reported in angiographic studies [11-13]. Given the rarity of this anomaly, the findings in this cadaveric case offer a unique opportunity for in-depth study, adding to the limited body of literature on this subject. 

Our literature search revealed only three cadaveric case reports of a double RCA. The first case described an anomalous aRCA that obliquely crossed the aorta and ended along the left margin of the left ventricle [11]. In contrast, our report displays an aRCA with a completely different course by descending on the anterior wall of the right ventricle, ending at the inferior margin near the apex of the heart. The second report described arteries with a similar course to the present case but did not mention the presence or absence of a conus branch or provide images depicting the anomalous arterial course [12]. In the third report of a double RCA, the aRCA coursed along the right side of the atrioventricular groove with the main RCA and terminated by forming the right marginal artery. Moreover, that report described a “shotgun” barrel shape to the septal orifice separating the RCA and aRCA [13]. This case differs because the aRCA does not give rise to the right marginal artery and has more of a carina-like shape to the septum separating the RCA and aRCA. The rarity and variability of this anomaly underscore the importance of recognizing such variations during diagnostic procedures and surgical interventions. 

Diagnostic catheterization of coronary arteries with anomalies has been reported to be more difficult [14,15]. Without prior imaging, such as coronary computed tomography angiography (CCTA), interventionalists may face difficulties navigating such variations. For example, in one case where a patient underwent catheterization of an anomalous RCA originating adjacent to the left coronary cusp, it was challenging to maneuver into the vessel due to its superior takeoff and anterior origin [14]. Similarly, with the anomaly seen in this case, procedural difficulties could arise due to the presence of the carina-like septum dividing the short trunk into two vessels within the common right coronary ostium. This septum artificially narrows the vessel and may complicate catheter insertion. This is consistent with one case report of double RCAs, which found that there is a possibility of missing the double RCA anomaly if the catheter is hooked deeply inside one of the RCAs with no or minimal reflux [9]. According to another study, a correct diagnosis of double RCA is not easily made based on conventional coronary angiography because it is difficult to distinguish this variation from that of a high takeoff of a large right ventricular branch [16]. One recent review of double RCAs indicated that complications could arise where the orifice at the bifurcation of double RCAs could act as a valve and cut off circulation to one of the arteries during filling [17]. These challenges highlight the need to diagnose and recognize coronary arteries with anomalies before catheterizations. 

The selection of an appropriate guiding catheter and preoperative imaging of the coronary arterial distribution could be significant. Catheter selection was important in some cases, especially if an anomalous RCA was involved in acute myocardial infarction (MI), as stenosis might have occurred in the proximal regions closer to its origin from the aorta [17,18]. In a study involving 185 patients with acute MI and atherosclerosis, eight patients had anomalous origin and occlusion at the proximal portion of the RCAs [19]. In this case, although the origin of the RCA from the right coronary sinus is not anomalous, the difficulty of inserting the catheter for patients with RCA occlusions could be increased by the septum dividing the main RCA and the aRCA within the ostium.

Moreover, the anomalous origin of the RCA (AAORCA) from the left sinus is one congenital anomaly that can have significant clinical implications. This anomaly can be particularly important because, when found, surgical repair by reimplantation or coronary artery bypass grafting (CABG) is recommended [20]. When performing the CABG procedure, the surgeon must be aware of the anomalous origin to correctly graft the new vessel and ensure adequate blood flow to the heart muscle [21]. If a CABG procedure was necessary for the anomaly described in this case report, the surgeon would need to bypass both the RCA and aRCA. Therefore, this example underscores the importance of preoperative imaging to identify possible coronary artery anomalies. Detailed imaging can, therefore, aid in preoperative surgical procedure planning to avoid complications and ensure optimal outcomes [20,21].

## Conclusions

This report presents a unique case of a double RCA. Most reported cases have been observed during angiography, which has certain limitations. With the cadaveric dissection in this case, we were able to demonstrate the details of the division of the main RCA and aRCA. Both vessels arose from a common ostium with an internal carina-like septum. Though rare, there can be significant clinical implications. Further research is needed to recognize the incidence and clinical significance of such a variation and help with preoperative planning to modify treatment strategies and improve patient outcomes.
